# The complex landscape of DMD mutations: moving towards personalized medicine

**DOI:** 10.3389/fgene.2024.1360224

**Published:** 2024-03-26

**Authors:** Francesca Gatto, Silvia Benemei, Giulio Piluso, Luca Bello

**Affiliations:** ^1^ Medical Affairs, Pfizer Italia, Rome, Italy; ^2^ Medical Genetics and Cardiomyology, Department of Precision Medicine, University of Campania “Luigi Vanvitelli”, Napoli, Italy; ^3^ Department of Neurosciences DNS, University of Padova, Padova, Italy

**Keywords:** Duchenne muscular dystrophy (DMD), dystrophin, genetic modifiers, genotype/phenotype, personalized medicine

## Abstract

Duchenne muscular dystrophy (DMD) is a severe genetic disorder characterized by progressive muscle degeneration, with respiratory and cardiac complications, caused by mutations in the DMD gene, encoding the protein dystrophin. Various DMD mutations result in different phenotypes and disease severity. Understanding genotype/phenotype correlations is essential to optimize clinical care, as mutation-specific therapies and innovative therapeutic approaches are becoming available. Disease modifier genes, trans-active variants influencing disease severity and phenotypic expressivity, may modulate the response to therapy, and become new therapeutic targets. Uncovering more disease modifier genes via extensive genomic mapping studies offers the potential to fine-tune prognostic assessments for individuals with DMD. This review provides insights into genotype/phenotype correlations and the influence of modifier genes in DMD.

## 1 Introduction

Duchenne muscular dystrophy (DMD) is a recessive genetic disorder characterized by progressive muscle degeneration caused by truncating mutations in the dystrophin gene (*DMD*), located on the X chromosome (Birnkrant et al., 2018; Duan et al., 2021). The disease, following an X-linked recessive inheritance pattern, primarily affects males. Females heterozygous for *DMD* mutations are usually asymptomatic carriers, although around 8% of female “manifesting carriers” are reported (Taylor et al., 2007; Duan et al., 2021).

The global prevalence of DMD has been estimated at 4.8 cases per 100,000 individuals, while in Italy it is estimated to be around 1.7–3.4 cases per 100,000 (Salari et al., 2022; Orso et al., 2023). DMD is one of the most severe types of muscular dystrophy with childhood onset (Ryder et al., 2017; Birnkrant et al., 2018; Duan et al., 2021). Skeletal muscle degeneration and subsequent muscle weakness in DMD typically begin early in life and progress over time, leading to motor delay, loss of ambulation, respiratory impairment, cardiac complications, and premature death (Birnkrant et al., 2018; Duan et al., 2021). Neurocognitive dysfunction may also be present in some children (Ricotti et al., 2016). The median life expectancy for patients with DMD born after 1990 is now around 30 years (Crisafulli et al., 2020; Broomfield et al., 2021), and the mean age at diagnosis in DMD is usually between 4 and 5 years (D'Amico et al., 2017; Ciafaloni et al., 2009; Thomas et al., 2022; Aartsma-Rus et al., 2019). A revision of DMD diagnosis in Italy between 2005 and 2014 identified a tendency to earlier diagnosis, with a mean age at diagnosis around 3.5 years (D'Amico et al., 2017).

Dystrophin is a cytoskeletal protein, crucial for the integrity of muscle fibers. It contains four major functional regions or domains: an N-terminal actin-binding domain, a rod domain consisting of 24 spectrin repeats interspersed by 4 “hinge” regions, a cysteine-rich domain which binds beta-dystroglycan, and a C-terminal domain mostly involved in the binding of signaling proteins (Duan et al., 2021). Dystrophin absence or severe deficiency leads to myofiber damage, cycles of fiber degeneration and regeneration, and eventually fibro-fatty substitution, resulting in progressive muscle weakness and loss of function (Duan et al., 2021; Mackenzie et al., 2021; Markati et al., 2022). A vast number of mutations have been reported in the dystrophin gene, the most frequent being large rearrangements, i.e., single-exon or multi-exon deletions, or more rarely duplications (Aartsma-Rus et al., 2006; Koeks et al., 2017). Mutations that disrupt the open reading frame (ORF) of the gene lead to dystrophin absence, and therefore to the severe DMD phenotype, while those that maintain an intact ORF, therefore allowing the expression of a defective protein, are associated with the milder Becker muscular dystrophy (BMD) phenotype (Monaco et al., 1988). Even within the spectrum of severe dystrophinopathy, different phenotypes in DMD have been distinguished (Humbertclaude et al., 2012), some mutational groups being associated with later loss of ambulation and milder respiratory involvement, sometimes defined as “intermediate” Duchenne/Becker muscular dystrophy (IMD) (Ferreiro et al., 2009; Gibbs et al., 2019; Zambon et al., 2022; van den Bergen et al., 2014; Bello et al., 2016a; Winnard et al., 1995; Wang et al., 2018; Torella et al., 2020).

Therapies aimed at restoring dystrophin expression are a focus of research. New disease-modifying, dystrophin-restorative therapies are emerging, representing the possibility of personalized molecular treatment for DMD, with the potential to slow disease progression and improve motor function in patients with specific mutations. The therapies aim to address the many needs arising from the primary muscle disease, together with adverse effects from long-term corticosteroid use as a component of current standard of care.

The introduction of mutation-specific therapies has made mutation identification and correct genetic diagnosis of critical importance in DMD, informing genetic counseling, assessment of carrier status and family planning and for assessing patient eligibility for novel molecular treatments. In fact, timely genetically-confirmed diagnosis is mandatory, as an early start of treatments has best chance of delivering a beneficial effect when initiated early in the disease course, before significant muscle degeneration and fibrosis have occurred (Aartsma-Rus et al., 2016; Bello and Pegoraro, 2016; Birnkrant et al., 2018; Aartsma-Rus et al., 2019; Duan et al., 2021).

In this review we will give an overview of the disease and gene mutations and discuss the personalized approaches to the treatment of DMD, with a focus on the underlying genotype/phenotype correlations.

## 2 Classification and frequency of mutations

### 2.1 Insights from the largest human gene and mutation patterns

The *DMD* gene is the largest known human gene, with 79 constitutive exons spanning approximately 2.5 million base pairs of genomic DNA (Muntoni et al., 2003; Aartsma-Rus et al., 2016). The large size of *DMD* makes it particularly prone to mutations, and thousands of different mutations have been identified in patients with DMD. Mutations can include large deletions, duplications, insertions, and point mutations (Aartsma-Rus et al., 2006; Ferreiro et al., 2009; Gibbs et al., 2019; Zambon et al., 2022; van den Bergen et al., 2014; Bello et al., 2016a; Winnard et al., 1995; Wang et al., 2018; Torella et al., 2020; Aartsma-Rus et al., 2016; Magri et al., 2011). The high mutation rate of *DMD* implies that about a third of DMD cases are the result of *de novo* mutation (Aartsma-Rus et al., 2016). Most patients have a deletion or duplication of single or multiple exons, but small mutations may also be present. Each type of mutation can lead to different functional consequences for the dystrophin protein. Certain mutations are amenable to mutation-specific therapies, and a number of gene-based therapeutic strategies are being developed or are under development, including exon skipping, stop codon read-through, vector-mediated gene therapy, and gene-editing strategies.

**FIGURE 1:**
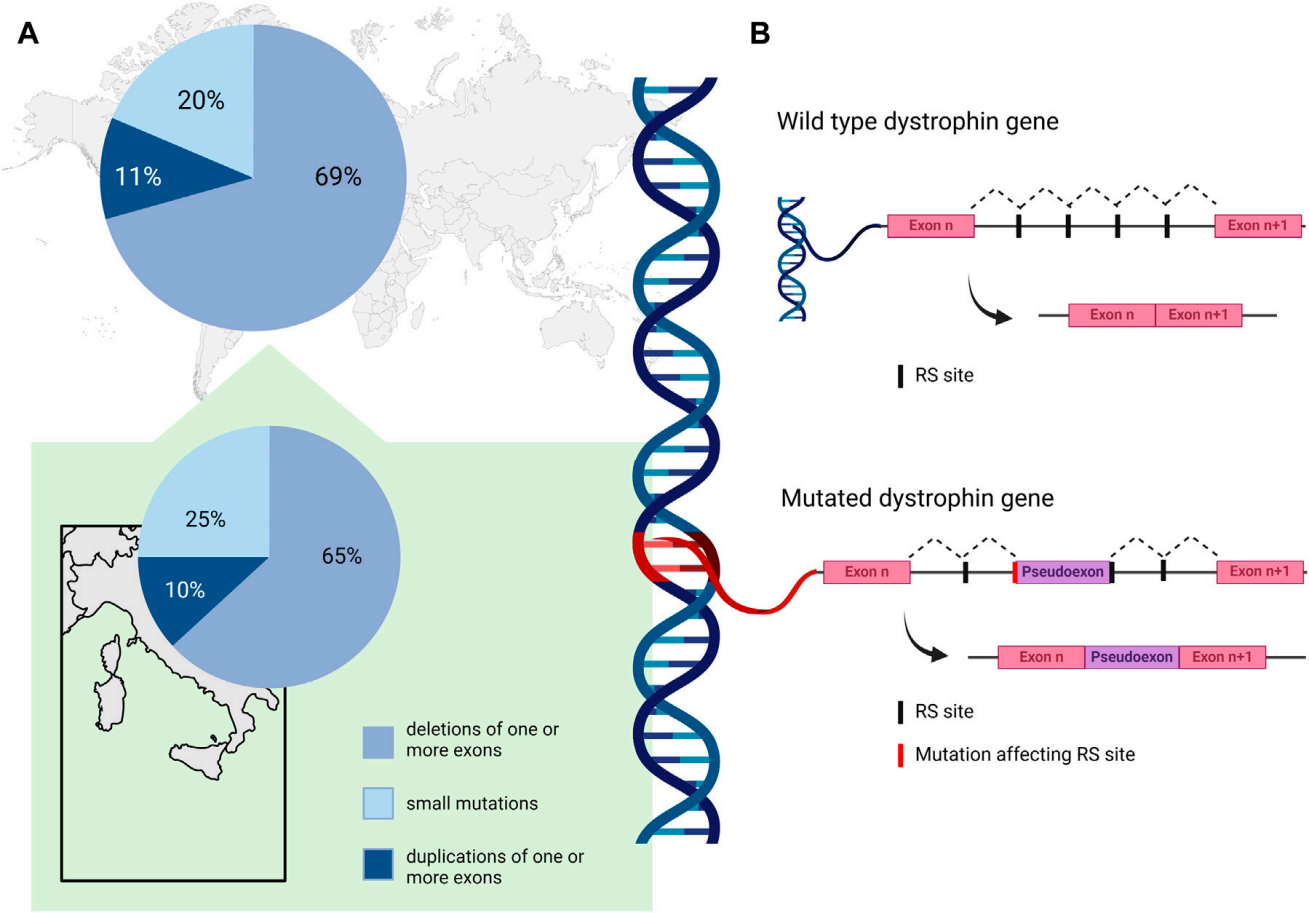
**(A)** Percent distribution of *DMD* mutation classes as reported by the TREAT-NMD DMD Global database (Bladen et al., 2015) (upper panel), and by an Italian nationwide study (Neri et al., 2020) (lower panel). **(B)**. Schematic representation of the non-canonical mechanism of recursive splicing recently observed in the pre-mRNA of the *DMD* gene. In the absence of mutation, recursive splicing correctly removes the intronic sequence from the mature transcript (wild type dystrophin gene). A deep intronic variant affecting a recursive intronic splice site (RS site) can cause the retention of a pseudoexon in the mature transcript of the gene (mutated dystrophin gene). Created with BioRender.com.

A comprehensive analysis of genetic data for 7,149 *DMD* mutations contained in the TREAT-NMD DMD Global database (http://umd.be/TREAT_DMD/) found that 80% of total mutations were large mutations, 69% of which were deletions and 11% duplications of one or more exons, while the remaining 20% were small mutations (Bladen et al., 2015) (Figure 1, upper panel A). Of the small mutations, 25% were small deletions, 9% small insertions, and 14% affected splice sites. Point mutations accounted for 52% of small mutations; 50% nonsense mutations and 2% missense mutations (Bladen et al., 2015).

### 2.2 Insights from the Italian population

In the Italian context, Neri and colleagues reported deletions in 65% of their nationwide cohort of 1,162 patients with DMD or BMD, while 10% had duplications and 25% small mutations (Neri et al., 2020) (Figure 1, lower panel A). Among the small mutations, which were distributed along the whole coding sequence, nonsense (11%) were the most frequent, followed by frameshifting small insertion-deletions (7%), while 4%, 1%, and 2% involved canonical splicing sites, splicing consensus, and rare missense mutations, respectively. Of note, some regional differences were described in the distribution of mutations in DMD patients; deletions were similarly frequent in Northern and Southern Italy (around 50%–80% in DMD and BMD patients), while they seem more frequent in Central Italy (around 85%–96% in DMD and BMD patients) (Neri et al., 2020). The distribution of duplications was again similar in Northern and Southern regions (around 10%–13%), and less frequent in Central regions (3%–7%).

Viggiano and colleagues, in their genetic analysis of 467 patients from Southern Italy with DMD, reported large deletions in 68%, while 12% had large duplications and 21% point mutations (Viggiano et al., 2023). The most frequently deleted exons were exons 48–50, 45–50, and 46–47, 46–48, and 49–50. Approximately a quarter of patients had deletions of only one exon, most frequently of involving exons 51, 44, or 45. The largest deletions were found in a “distal hotspot” region of the *DMD* gene (exons 45–55), where deletions have been shown to cluster preferentially (Aartsma-Rus et al., 2006; Duan et al., 2021; Andrews et al., 2022). The largest duplication involved 37 exons from 33 to 60, while single exon duplications involved exons 2, 12, 44, 50, 51, 52, 54, or 56. The exons most frequently duplicated were 2 and 44, with duplications typically occurring at the 5′end in exons 2–23 (“proximal hotspot”), and less frequently at the 3′end in exons 44–60. Point mutations, randomly distributed along the *DMD* gene without preferential hotspots, were identified in 21% of patients (Viggiano et al., 2023).

### 2.3 Deep intronic variants and recursive splicing mechanisms

Elusive pathogenic mutations in the *DMD* gene may be due to deep intronic variants that can cause aberrant splicing and introduce pseudo-exons (PEs) into the dystrophin coding sequence (Tuffery-Giraud et al., 2003; Zaum et al., 2017; Keegan, 2020; Waldrop et al., 2022; Segarra-Casas et al., 2023). They can be identified by RNA analysis and a multi-omics approach for their effect on the transcript. Deep intronic variants can affect pre-mRNA splicing by activating cryptic intronic acceptor or donor sites, causing PE inclusion, and by altering regulatory sequence motifs recognized by specific RNA binding proteins (Vaz-Drago et al., 2017). Although their frequency in DMD patients has been estimated to be about 0.3% (Bladen et al., 2015), this class of pathogenic variants is most likely underestimated (Waldrop et al., 2022). For the removal of long introns, such as in *DMD*, a non-canonical mechanism of recursive splicing, first described in *Drosophila* (Burnette et al., 2005), has recently been observed in the dystrophin pre-mRNA (Gazzoli et al., 2016). These recursive intronic splice sites (RS sites) contain a 3′splice site immediately followed by a sequence corresponding to a 5′splice site (Sibley et al., 2015), and it is possible that deep intronic variants affecting RS sites could contribute to PE activation in *DMD* (Keegan, 2020). The non-canonical mechanism of recursive splicing, recently observed in pre-mRNA of *DMD* gene, is schematized in Figure 1 panel B. A deep intronic variant affecting a RS site can cause the retention of a pseudoexon in the mature transcript of the gene.

Overall, the “genetic architecture” of DMD seems similar across world populations, with a vast allelic heterogeneity (i.e., thousands of pathogenetic mutations), a preponderance of large deletions, most frequently clustered at mutational hotspots, and a high rate of *de novo* mutations. As a rule, there are no “founder” mutations with high allelic frequency in specific world populations, with very few exceptions (Flanigan et al., 2009), and regional differences described in the studies reviewed above should be taken with caution, as they may be at least in part attributed to ascertainment bias (e.g., availability of sequencing), to familial clusters, or to chance.

## 3 Genotype/phenotype correlations

Characterizing mutations in individual patients, and ascertaining their frequency in *DMD* populations, is an invaluable tool for advancing basic scientific research on DMD-causing mutations, and in determining the accurate genetic diagnosis necessary to optimize clinical care.

Multiplex ligation-dependent probe amplification (MLPA) can screen all 79 *DMD* exons for deletions and duplications, but is unable to detect small mutations, whereas next-generation sequencing (NGS) techniques provide a more precise method of detecting and elucidating small mutations in neuromuscular diseases. With increasing efficacy, high coverage whole exome sequencing (WES) and especially whole genome sequencing (WGS) may also be employed for copy number variant analysis, and therefore identify large deletions and duplications, enabling more reliable and accurate genetic diagnosis (Stockley et al., 2006; Aartsma-Rus et al., 2009; Volk and Kubisch, 2017; Ebrahimzadeh-Vesal et al., 2018; Sheikh and Yokota, 2020). “Older” analytical methods, such as Sanger sequencing, multiplex polymerase chain reaction, and comparative genome hybridization array, also remain in use for confirming DMD in certain contexts.

Basic research studies into the pathogenesis of DMD have added to the understanding of the relationship between dystrophin structure and function, and provided some indication of dystrophin isoforms connected with muscle involvement, cognitive impairment, or cardiac disease. For instance, studies suggest that individual dystrophin isoforms are specific to or expressed at higher levels in skeletal and cardiac muscle (Dp427m), brain and CNS (Dp427c), the retina (Dp260), the central nervous system and kidney (Dp140), the peripheral nerves and Schwann cells (Dp116), and brain, liver, and cardiac muscle (Dp71) (Muntoni et al., 2003; Duan et al., 2021). Dp427p has been described in the Purkinje cells (whence the “p” designation) in the murine cerebellum, but expression studies in humans find it virtually absent from the CNS (Doorenweerd et al., 2017). Altogether, these isoforms influence early aspects of gross motor and neurocognitive development (Lim et al., 2020; Duan et al., 2021; Norcia et al., 2021).

Documenting genotype/phenotype relationships is essential to guide mutation-specific therapies. It is clear that different patterns of exon deletions or duplications can impact the severity of symptoms and signs, hence determining the phenotype of the disease. Patients with deletions of specific exons may exhibit a milder phenotype compared to those with larger deletions involving multiple exons. With some exceptions, the size or location of deletions or duplications leading to out-of-frame mutations do not normally affect the clinical phenotype, as no functional protein is produced, resulting in severe DMD phenotypes (Magri et al., 2011; Aartsma-Rus et al., 2016; Duan et al., 2021). Out-of-frame mutations usually lead to the total absence of functional dystrophin, whereas in-frame mutations may allow the production of partially functional dystrophin, leading to milder phenotypes like BMD (Aartsma-Rus et al., 2006; Aartsma-Rus et al., 2016; Anwar et al., 2021). However, approximately 10% of genetic mutations are exceptions to the reading frame rule, and some patients with in-frame mutations may present with severe DMD, while patients with out-of-frame mutations may turn out to have a milder BMD phenotype. The frequencies of such exceptions have been estimated as around 10% in BMD, and 5% in DMD, with exceptions occurring more frequently at the 5’ end of the gene (Aartsma-Rus et al., 2006; Kesari et al., 2008).

Examples of well-established genotype-phenotype correlations within the DMD spectrum include several mutations linked to milder DMD, or IMD, such as deletion of exons 3–7 (Muntoni et al., 1994; Winnard et al., 1995; Gualandi et al., 2006; Bello et al., 2016a) or other deletions bordering exon 8 (Wang et al., 2018). These mutations may be rescued by downstream translational reinitiation from an alternative ATG codon, a mechanism probably also shared by exon 2 duplication (Zambon et al., 2022) and proximal nonsense mutations (Torella et al., 2020). Additionally, deletions bordering exon 44 are observed (van den Bergen et al., 2014; Bello et al., 2016a; Pane et al., 2014), probably due to alternative splicing of this exon, leading to low levels of an in-frame transcript (Dwianingsih et al., 2014; Coratti et al., 2021; Muntoni et al., 2023).

Conversely, mutations bordering (and therefore amenable to skipping of) exon 51 and 53 are considered to be linked to especially severe DMD phenotypes (Coratti et al., 2021; Muntoni et al., 2023).

## 4 Genetic modifiers

Disease severity related to a particular mutation may vary quite substantially, at times even between members of the same family, with modulation by modifier genes that interact with DMD pathophysiological events (Vo and McNally, 2015). Identifying these modifiers can help explain differences in the disease presentation among individuals with the same DMD mutation; furthermore, identified modifiers may in themselves become targets for therapeutic interventions.

**FIGURE 2:**
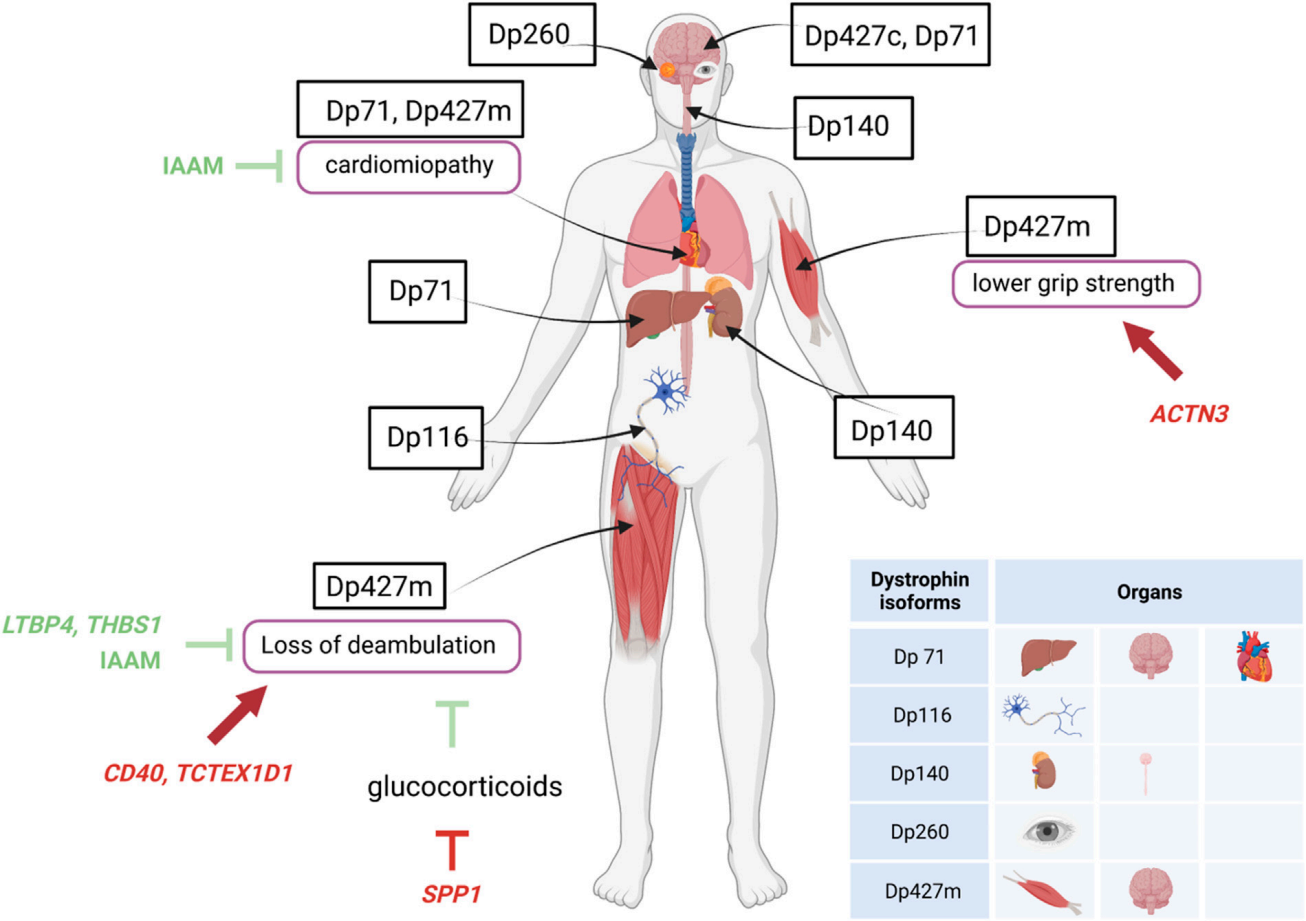
Specific expression of dystrophin isoforms and modulation of DMD severity by genetic modifiers. Figure shows the localization of the different dystrophin isoforms within cells or organs and the influence of genetic modifiers (variants of genes unrelated to dystrophin) in either protecting (green lines) or worsening (red lines) DMD severity. *ACTN3*, actinin-3 gene; IAAM, Isoleucine-Alanine-Alanine-Methionine haplotype; *LTBP4S*, Latent Transforming growth factor β Binding Protein 4 gene; *SPP1*, osteopontin gene; *TCTEX1D1*, Tctex1 domain containing 1 gene; *THBS1*, thrombospondin-1 gene.

Genetic modifiers may be defined as trans-active variants, i.e., polymorphisms in genes remote from the disease-causing *DMD* gene, that may influence disease severity, expressivity of disease phenotypes and sub-phenotypes (e.g., cardiac, respiratory), or response to treatments (Figure 2). Several such variants have been described in DMD, and are thoroughly reviewed elsewhere (Bello and Pegoraro, 2019; Bello et al., 2023).

A single nucleotide polymorphism (SNP) in the promoter of the *SPP1* gene, encoding the pleiotropic cytokine osteopontin, was the first variant to be associated with DMD severity, and more specifically to earlier loss of ambulation (LoA) and lower grip strength values (Pegoraro et al., 2011). This observation was validated in some independent DMD cohorts (Bello et al., 2012; Bello et al., 2015; Chen et al., 2020), but not in others (Flanigan et al., 2013; Van Den Bergen et al., 2015), possibly due to a role of osteopontin in the modification of response to glucocorticoid treatment, rather than DMD severity *per se*; a phenomenon that was also investigated *in vitro* (Barfield et al., 2014; Vianello et al., 2017). Importantly, the detrimental *SPP1* genotype, while apparently predisposing to a reduced response to glucocorticoids, does in no way contraindicate treatment. Osteopontin is implicated in muscle damage and regeneration, acting both as a pro-inflammatory cytokine in the acute phase of damage (Vetrone et al., 2009) and a scaffold for regenerating myotubes during repair (Uaesoontrachoon et al., 2012; Pagel et al., 2014). All of these mechanisms are relevant in the degeneration/regeneration cycles ensued by dystrophin deficiency, and their attenuation by glucocorticoid treatment.

Subsequently, a haplotype of four coding SNPs in the gene encoding Latent Transforming growth factor β Binding Protein 4 (*LTBP4*) was associated to age at LoA in severe dystrophinopathy (Flanigan et al., 2013). Again, the association was validated independently (Bello et al., 2015; Van Den Bergen et al., 2015), although not in all studied populations (Barp et al., 2015; Chen et al., 2020; Kosac et al., 2022). The homozygote state for the protective Isoleucine-Alanine-Alanine-Methionine (IAAM) haplotype was associated to later LoA, and the resulting isoprotein is predicted to give rise to a stable latent complex with TGF-β, which prevents this potent pro-fibrotic cytokine from interacting with its cell surface receptors (Ceco et al., 2014). This anti-fibrotic action of the IAAM haplotype of *LTBP4* may also explain its apparent protective function from the onset and progression of *DMD*-related cardiomyopathy (Barp et al., 2015; Bello et al., 2023).

Following the first two described modifiers, several other loci have been linked to modulation of DMD severity, including *CD40*, a signaling molecule involved in the transition from innate to specific immunity and the modulation of pro-inflammatory (M1) *versus* pro-regenerative (M2) macrophage pools (Bello et al., 2016b); *ACTN3*, encoding the sarcomeric protein actinin-3, which is specific to fast-twitch muscle fibers and is missing because of a common nonsense SNPs in 18% of healthy individuals (Hogarth et al., 2017); *TCTEX1D1*, encoding a protein with scarce functional annotations, but emerging from a WES scan of extremely severe DMD patients (Spitali et al., 2020); *THBS1*, encoding thrombospondin-1, a protease activator of the LTBP/TGF-β complex (Weiss et al., 2018).

The described effect size of genetic modifiers is generally smaller than that of the specific *DMD* mutations described above (i.e., del 3-7, deletions bordering exon 44), and do not allow strong prognostic predictions in individual patients. However, they may be of use in post-hoc analyses of observational and interventional cohort studies in DMD, allowing a better resolution and interpretation of the variability observed in outcomes (Bello et al., 2023). Many of the described modifiers focus on pathways implicated in muscle inflammation, regeneration, and fibrosis, highlighting the importance of these mechanisms in the downstream effects of dystrophin deficiency (Bello and Pegoraro, 2019). More modifiers may yet be undiscovered; their full characterization, which may be attained by large scale genomic mapping studies in collaborative international DMD cohorts, may allow the identification of novel therapeutic targets, and, through the implementation of multi-locus interaction models, to improved genetic counseling and prognosis for the DMD population.

## 5 Personalized treatments

**FIGURE 3:**
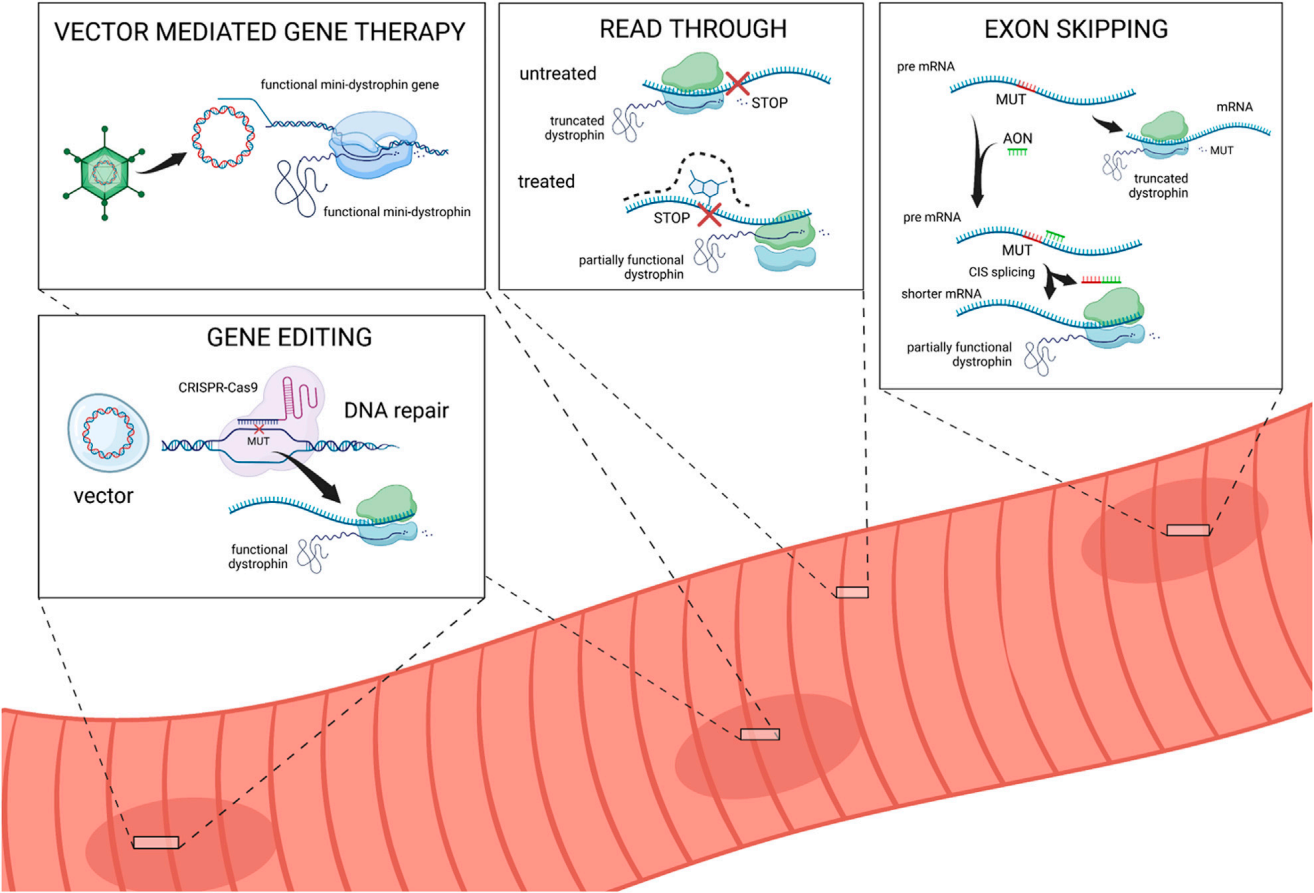
Schematic overview of DMD therapeutic approaches. Vector mediated gene therapy: adeno-associated viral vectors deliver mini-dystrophin gene to muscle cells. After binding to the cell membrane, the vector delivers its content which is maintained as an episome in the host cell nucleus, leading to the expression of the therapeutic protein. Read-through therapy: nonsense mutations in the *DMD* gene introduce premature stop codons resulting in the synthesis of truncated, non-functional dystrophin. The read-through of the premature stop codon achieved by drugs allows for the continued translation process yielding at least partly functional dystrophin. Exon skipping: mutations in the coding sequence disrupt the translational reading frame of the gene. Interruption or deletion of coding regions result in truncated or non-functional dystrophin. Antisense oligoribonucleotide (AON) selectively “skips” specific exons aiming to restore the reading frame of the gene, facilitating the production of a partially functional dystrophin protein. Gene editing: CRISPR/Cas9-mediated gene editing allows the specific modification of a target sequence. The repaired DNA can be translated in a fully functional protein. Created with BioRender.com.

Although no definitive cure is available for DMD, mutation-specific therapies may target individual mutations, and innovative therapeutic approaches have been developed over recent decades or are undergoing clinical investigation. The most researched of these approaches include exon skipping, vector-mediated gene therapy, stop codon read-through, and gene-editing strategies. Gene-based therapeutic strategies targeting dystrophin have the potential to deliver durable benefits in DMD with one-time treatment (Yao et al., 2021) (Figure 3).

### 5.1 Exon skipping therapy

Antisense oligoribonucleotide (AON)-mediated exon skipping therapies aimed at restoring the reading frame and gene replacement therapies have emerged as promising treatments and are being investigated in clinical trials. Exon skipping aims to “skip”, i.e., exclude from being spliced into the mature messenger RNA, specific exons bordering out-of-frame deletions in the *DMD* gene, thereby restoring the ORF and allowing the production of a truncated but partially functional dystrophin protein, analogous with that typically found in patients with BMD (Bello and Pegoraro, 2016). This approach is currently applied only to deletions and can be tailored to the individual’s specific mutations, making it a personalized treatment option.

The peculiar pathogenetic mechanism of the deep intronic variant that introduces PEs in the *DMD* transcript may be of particular interest for more targeted and personalized therapies. In fact, while currently available and experimental therapeutic strategies may only lead to a more functional dystrophin with minimal regression of disease severity, antisense oligonucleotide-mediated PE skipping may be a promising precision medicine strategy that can potentially transform a DMD/BMD phenotype into a healthy one (Gurvich et al., 2008; Rimessi et al., 2010; Enkhjargal et al., 2023).

Several versions of AONs, such as phosphorodiamidate morpholino oligomers (PMO) compounds were developed, including molecules designed to skip exon 51 (Charleston et al., 2018; McDonald et al., 2021), exon 53 (Frank et al., 2020; Clemens et al., 2023) and exon 45 (Wagner et al., 2021). These therapeutic molecules have received regulatory approval by the US Food and Drug Administration (FDA), but European Medicines Agency (EMA) approval awaits long-term safety and efficacy data.

Weekly intravenous infusions of PMO AONs are generally safe and well tolerated in DMD patients at high dosing levels. However, the long-term effectiveness of PMO exon skipping therapies in delaying DMD progression have not been established, and their clinical usefulness is limited due to sub-optimal tissue targeting resulting in low levels of dystrophin restoration in skeletal and cardiac muscle (Fortunato et al., 2021). Improvements to PMO technology, such as conjugation with cell-penetrating peptides (Lim et al., 2022), or delivery through receptor-based platforms (Desjardins et al., 2022), may allow increased efficacy and hopefully translate into higher dystrophin restoration levels and clinical benefit.

### 5.2 Vector-mediated gene therapy

Recent advances in gene replacement therapy approaches that involve delivering a functional version of the *DMD* gene into muscle cells using adeno-associated virus (AAV) vectors have shown promising results in preclinical and clinical trials, prompting optimism that a potential treatment or long-term improvement of DMD may be possible. However, substantial obstacles remain. In particular, the large size of the full-length *DMD* transcript in relation to the carrying capacity of the AAV vector has necessitated the use of shortened transgenes that code for mini- and micro-dystrophin proteins (Barthelemy and Wein, 2018; Grages et al., 2020; Fortunato et al., 2021). However, as with other gene-based therapies, pre-existing immunity, potential immune responses towards both the vector capsid and microdystrophin itself, duration of response, and the inability of re-dosing are issues that must be addressed (Elangkovan and Dickson, 2021; Fortunato et al., 2021)**.**

Delandistrogene moxeparvovec-rokl (Sarepta Therapeutics) is a recombinant vector-mediated gene transfer therapy containing a micro-dystrophin transgene. Following a randomized controlled trial that confirmed expression and correct localization of micro-dystrophin after administration, suggestive of potential clinical benefit (Mendell et al., 2023; Zaidman et al., 2023), delandistrogene moxeparvovec received accelerated approval by the FDA for use in ambulatory children with a confirmed mutation in the *DMD* gene (U. S. Food & Drug Administration FDA, 2023; Hoy et al., 2023). Further research into the therapy continues in several confirmatory trials.

Other AAV-mediated gene therapies that have been investigated or are under active development include SGT-001 (NCT03368742) and PF-06939926 (NCT03362502, NCT04281485) (Barthelemy and Wein, 2018; Elangkovan and Dickson, 2021).

Of particular interest are the analogies and differences in the design of micro-dystrophins brought on in different programs (McGreevy et al., 2015). All designs retain the essential N-terminal (actin-binding) and cysteine-rich (β-dystroglycan-binding) domains, as well as the spectrin repeats adjacent to those respective domains, i.e., the first (R1) and the last (R24); and all dispense with the C-terminal domain, which has signaling rather than structural functions. On the other hand, differences include: the total number of spectrin repeats included (4 or 5, out of the 24 of wild-type dystrophin); the choice of spectrin repeats other than 1 and 24, only one construct including the neuronal nitric oxide synthase (nNOS) binding repeats 16 and 17; and the choice of hinge domains, which confer flexibility to the rod structure of dystrophins (although all micro-dystrophins contain three hinges, as opposed to the four hinges of the full-length protein).

Possible concern for this treatment is due to the T-cell immune response observed upon AAV-delivered micro-dystrophin, as some patients involved in three different trials showed an immune response directed against peptide pools within exons 8–11, nonself epitopes within the micro-dystrophin construct (Bonnemann et al., 2023). Ongoing research is addressed to better understand the specific regions involved in the immune response to provide adjusted immunomodulation protocols ensuring the safety of the treatment.

### 5.3 Read-through therapy

Nonsense mutation-related premature stop codons in the *DMD* gene sequence prematurely terminating the translation of dystrophin during protein synthesis, result in truncated and dysfunctional dystrophin protein (Fortunato et al., 2021) and/or degradation of the transcript through nonsense-mediated decay. Promotion of read-through of the premature stop codon has the potential to suppress the premature stop signal, leading to production of at least partly functional dystrophin (Fortunato et al., 2021). An oral drug, ataluren (PTC-124, PTC Therapeutics) designed to bind ribosomal RNA subunits to impede the recognition of stop codons, was developed and received regulatory approval in Europe in 2014 (Fortunato et al., 2021), for ambulatory patients aged 5 years and older, and was later extended to 2 and older. Dystrophin restoration data in patient muscle biopsies are less well characterized than for exon skipping and gene replacement therapies, but indicate some level of dystrophin restoration (Finkel et al., 2013). Ataluren has shown clinical benefits, such as a slowing of disease progression as measured by the 6 min walk test and North Star Ambulatory Assessment (NSAA). Although it failed to meet primary study endpoints in three placebo-controlled studies (Bushby et al., 2014; McDonald et al., 2017; McDonald et al., 2023), it did demonstrated efficacy throughout a series of outcomes, especially in specific sub-groups, such as patients with an “intermediate” (i.e., not too mildly, nor too severely impaired) ambulatory function. A post-marketing registry of patients treated with commercial ataluren has confirmed a reassuring safety profile as well as suggested prolongation of independent ambulation in treated patients compared to “natural history” controls treated with standards of care (Mercuri et al., 2020). In September 2023 EMA recommended against renewing the authorization for ataluren (European Medicines Agency EMA, 2023), decision confirmed on 26 January 2024 following re-examination of data of a post-authorization study and of data comparing two patients registries (European Medicines Agency, 2024).

### 5.4 Gene editing

Editing the genome to permanently correct genetic effects is emerging as a promising therapeutic approach in DMD. The most advanced of the gene editing methods being studied for DMD is CRISPR/Cas9. CRISPR/Cas9-mediated gene editing systems provide effective, specific, and versatile technologies utilizing programmable nucleases, and have transformed basic science research while offering enormous potential for individualized treatment of a broad range of DMD mutations, including single- or multi-exon deletions (Fortunato et al., 2021). The technology allows the generation of a range of genomic variations in the target region, including deletions, insertions, and substitutions, designed to correct, interrupt, or eliminate gene defects (Bello and Pegoraro, 2016; Fortunato et al., 2021; Happi Mbakam et al., 2022). The specific mutation and DNA sequence of each patient allow a great flexibility in selecting the target site for gene editing, according to the specific purpose. Nevertheless, in some cases, there may be limited target DNA sequence, reducing the chance for an effective editing of the CRISPR/Cas9 system (Happi Mbakam et al., 2022). In addition, delivery approaches should be implemented to specifically target different tissues (Happi Mbakam et al., 2022).

There is preclinical evidence that CRISPR/CAS9 systems can reframe mutated *DMD*, potentially allowing dystrophin restoration, although the technology is yet to be demonstrated successfully in patients. Of note, a recent case report details an unforeseen event in a patient with DMD who experienced acute respiratory distress and cardiac arrest, leading to a fatal outcome at day 8 following the delivery of CRISPR/CAS9 via AAV9 (Lek et al., 2023).

The anti-Cas9 immune response is a significant challenge for gene editing application by limiting the safety and efficacy of therapies, leading to unfavorable immune reaction (Crudele and Chamberlain, 2018; Ewaisha and Anderson, 2023).

Thus, issues remain to be addressed, including targeting specificity, possible off-target mutagenetic effects resulting in genome instability or genotoxicity, optimal delivery of the gene editing components, and potential immune reactions (Choi and Koo, 2021; Duan et al., 2021; Elangkovan and Dickson, 2021; Fortunato et al., 2021). Gene editing techniques need to be implemented before the use in DMD patients.

### 5.5 Other approaches

Other therapeutic approaches include cell therapy targeted to dystrophin replacement or repair. For example, stem cells containing a functional copy of *DMD* genetically modified *in vitro* from the patient’s own cells (autologous transplantation), or already functional, dystrophin-competent cells sourced from a donor (allogeneic transplantation) have the potential to effect muscle repair upon transplantation into affected muscle (Barthelemy and Wein, 2018; Grages et al., 2020; Duan et al., 2021; Fortunato et al., 2021; Markati et al., 2022). However, cell survival and migration into damaged target muscle in the host is limited, and restoration of dystrophin expression may be transient. Furthermore, the need of arterial injection poses several practical difficulties, not only due to procedural risks, but also because of the difficulty to reach crucially important axial, respiratory, and cardiac muscles.

Upregulation of utrophin, a dystrophin surrogate protein, is another potential therapeutic strategy under investigation, as the gene encoding utrophin is not mutated and may be able to deliver a degree of functional redundancy at the sarcolemma during muscle development (Grages et al., 2020).

Lastly, allogeneic cardiosphere-derived cell (CDCs) therapy is being investigated as a potential regenerative treatment for DMD. CDCs are stromal cells secreting exosomes which fuse with macrophages and fibroblasts, delivering information to “reprogram” the target cell. As a result, macrophages turn from a pro-inflammatory to a protective phenotype and fibroblasts revert from fibrotic to antifibrotic phenotype slowing disease progression (de Couto et al., 2015; Tseliou et al., 2015; McDonald et al., 2022).

## 6 Discussion

Individual mutations in the dystrophin gene provide the opportunity for mutation-specific targeted personalized therapies to slow DMD progression or revert DMD into a milder phenotype (Bello and Pegoraro, 2016). The emerging evidence suggests that personalized treatments for DMD have the potential to increase dystrophin production and improve motor function in patients with specific mutations, slowing disease progression or resulting in leading towards the milder phenotype of BMD (Fortunato et al., 2021). In this landscape, genetic assessment emerges as a pivotal tool, enabling the identification of individual mutations necessary to guide the application of targeted therapies. As molecular treatments aimed at dystrophin restoration in DMD are increasingly available as commercialized drugs or within clinical trials, genetic diagnosis has become an indispensable tool in order to determine eligibility for these treatments, which can reduce disease progression and improve the quality of life for affected individuals.

To better understand which patients are amenable to mutation-specific therapies, patient data must be collected broadly through studies and registries (https://www.dmd.nl/; http://umd.be/TREAT_DMD/). Registries provide a source of information for understanding the disease, its management, the efficacy and safety in the long-term, and the cost-effectiveness of new therapies (Koeks et al., 2017). Indeed, through documenting the genotype–phenotype relationship, researchers may better design mutation-specific therapies, ensuring they are precisely tailored to individual needs, for the selection of the treatment with respect to the feature of the single patient. The potential long-term benefits of these innovative therapies can significantly improve the QoL of DMD patients (Schwartz et al., 2023).

The ongoing research and progress in personalized treatments promises a more positive future for individuals with DMD. Continued efforts to better understand genotype/phenotype correlations with the collection of detailed neuromuscular disease natural history data, developing relevant patient and mutation-specific models (Bartoli et al., 2023), and expanding treatment options can be expected to significantly impact the lives of those affected by this debilitating condition. However, despite the significant progress in mutation-specific therapies for DMD, challenges in developing personalized treatments for DMD remain, including cost, ensuring widespread accessibility, possible vector-associated immune responses, long-term efficacy, the duration of responses, and whether re-dosing will be required to maintain benefits.

Guidance from regulatory agency is needed in this direction, pointing to personalized assessment and mitigation strategies that can be implemented for individuals receiving genetic medicine approaches, including screening for immune responses and engineering proteins to silence immunodominant epitopes (Ewaisha and Anderson, 2023).

Questions also remain as to how genetic modifiers can influence the efficacy of genetic approaches such as exon skipping, stop codon read-through, and vector-mediated gene therapy.

## References

[B1] Aartsma-Rus A. Fokkema I. Verschuuren J. Ginjaar I. van Deutekom J. van Ommen G. J. (2009). Theoretic applicability of antisense-mediated exon skipping for Duchenne muscular dystrophy mutations. Hum. Mutat. 30 (3), 293–299. 10.1002/humu.20918 19156838

[B2] Aartsma-Rus A. Ginjaar I. B. Bushby K. (2016). The importance of genetic diagnosis for Duchenne muscular dystrophy. J. Med. Genet. 53 (3), 145–151. 10.1136/jmedgenet-2015-103387 26754139 PMC4789806

[B3] Aartsma-Rus A. Hegde M. Ben-Omran T. Buccella F. Ferlini A. Gallano P. (2019). Evidence-based consensus and systematic review on reducing the time to diagnosis of Duchenne muscular dystrophy. J. Pediatr. 204, 305–313. 10.1016/j.jpeds.2018.10.043 30579468

[B4] Aartsma-Rus A. Van Deutekom J. C. Fokkema I. F. Van Ommen G. J. Den Dunnen J. T. (2006). Entries in the Leiden Duchenne muscular dystrophy mutation database: an overview of mutation types and paradoxical cases that confirm the reading-frame rule. Muscle Nerve 34 (2), 135–144. 10.1002/mus.20586 16770791

[B5] Andrews J. G. Lamb M. M. Conway K. M. Street N. Westfield C. Ciafaloni E. (2022). Differentiation of pediatric-onset duchenne and becker muscular dystrophy subphenotypes using data from the muscular dystrophy surveillance tracking and research network (MD STARnet). J. Neuromuscul. Dis. 9 (1), 171–178. 10.3233/JND-210739 34776418 PMC9059491

[B6] Anwar S. He M. Lim K. R. Q. Maruyama R. Yokota T. (2021). A genotype-phenotype correlation study of exon skip-equivalent in-frame deletions and exon skip-amenable out-of-frame deletions across the DMD gene to simulate the effects of exon-skipping therapies: a meta-analysis. J. Pers. Med. 11 (1), 46. 10.3390/jpm11010046 33466756 PMC7830903

[B7] Barfield W. L. Uaesoontrachoon K. Wu C. S. Lin S. Chen Y. Wang P. C. (2014). Eccentric muscle challenge shows osteopontin polymorphism modulation of muscle damage. Hum. Mol. Genet. 23 (15), 4043–4050. 10.1093/hmg/ddu118 24626632 PMC4082368

[B8] Barp A. Bello L. Politano L. Melacini P. Calore C. Polo A. (2015). Genetic modifiers of Duchenne muscular dystrophy and dilated cardiomyopathy. PLoS One 10 (10), e0141240. 10.1371/journal.pone.0141240 26513582 PMC4626372

[B9] Barthelemy F. Wein N. (2018). Personalized gene and cell therapy for Duchenne muscular dystrophy. Neuromuscul. Disord. 28 (10), 803–824. 10.1016/j.nmd.2018.06.009 30224293

[B10] Bartoli M. Bailey R. M. Meyer K. Barthelemy F. (2023). Editorial: personalized medicine for neuromuscular disorders. Front. Cell Dev. Biol. 11, 1329048. 10.3389/fcell.2023.1329048 38033860 PMC10687627

[B11] Bello L. Sabbatini D. Fusto A. Gorgoglione D. Borin G. U. Penzo M. (2023). The IAAM LTBP4 Haplotype is Protective Against Dystrophin-Deficient Cardiomyopathy. J. Neuromuscul. Dis. 10.3233/JND-230129 PMC10977446 38363615

[B12] Bello L. Flanigan K. M. Weiss R. B. Dunn D. M. Swoboda K. J. Gappmaier E. (2016b). Association study of exon variants in the NF-κB and TGFβ pathways identifies CD40 as a modifier of Duchenne muscular dystrophy. Am. J. Hum. Genet. 99 (5), 1163–1171. 10.1016/j.ajhg.2016.08.023 27745838 PMC5097949

[B13] Bello L. Hoffman E. P. Pegoraro E. (2023). Is it time for genetic modifiers to predict prognosis in Duchenne muscular dystrophy? Nat. Rev. Neurol. 19 (7), 410–423. 10.1038/s41582-023-00823-0 37308617

[B14] Bello L. Kesari A. Gordish-Dressman H. Cnaan A. Morgenroth L. P. Punetha J. (2015). Genetic modifiers of ambulation in the cooperative international Neuromuscular research group Duchenne natural history study. Ann. Neurology 77 (4), 684–696. 10.1002/ana.24370 PMC4403971 25641372

[B15] Bello L. Morgenroth L. P. Gordish-Dressman H. Hoffman E. P. McDonald C. M. Cirak S. (2016a). DMD genotypes and loss of ambulation in the CINRG duchenne natural history study. Neurology 87 (4), 401–409. 10.1212/WNL.0000000000002891 27343068 PMC4977110

[B16] Bello L. Pegoraro E. (2016). Genetic diagnosis as a tool for personalized treatment of Duchenne muscular dystrophy. Acta Myol. 35 (3), 122–127. 28484312 PMC5416739

[B17] Bello L. Pegoraro E. (2019). The "Usual Suspects": genes for inflammation, fibrosis, regeneration, and muscle strength modify Duchenne muscular dystrophy. J. Clin. Med. 8 (5), 649. 10.3390/jcm8050649 31083420 PMC6571893

[B18] Bello L. Piva L. Barp A. Taglia A. Picillo E. Vasco G. (2012). Importance of SPP1 genotype as a covariate in clinical trials in Duchenne muscular dystrophy. Neurology 79 (2), 159–162. 10.1212/WNL.0b013e31825f04ea 22744661 PMC3390537

[B19] Birnkrant D. J. Bushby K. Bann C. M. Apkon S. D. Blackwell A. Brumbaugh D. (2018). Diagnosis and management of Duchenne muscular dystrophy, part 1: diagnosis, and neuromuscular, rehabilitation, endocrine, and gastrointestinal and nutritional management. Lancet Neurol. 17 (3), 251–267. 10.1016/S1474-4422(18)30024-3 29395989 PMC5869704

[B20] Bladen C. L. Salgado D. Monges S. Foncuberta M. E. Kekou K. Kosma K. (2015). The TREAT-NMD DMD Global Database: analysis of more than 7,000 Duchenne muscular dystrophy mutations. Hum. Mutat. 36 (4), 395–402. 10.1002/humu.22758 25604253 PMC4405042

[B21] Bonnemann C. G. Belluscio B. A. Braun S. Morris C. Singh T. Muntoni F. (2023). Dystrophin immunity after gene therapy for duchenne's muscular dystrophy. N. Engl. J. Med. 388 (24), 2294–2296. 10.1056/NEJMc2212912 37314712

[B22] Broomfield J. Hill M. Guglieri M. Crowther M. Abrams K. (2021). Life expectancy in Duchenne muscular dystrophy: reproduced individual patient data meta-analysis. Neurology 97 (23), e2304–e2314. 10.1212/WNL.0000000000012910 34645707 PMC8665435

[B23] Burnette J. M. Miyamoto-Sato E. Schaub M. A. Conklin J. Lopez A. J. (2005). Subdivision of large introns in Drosophila by recursive splicing at nonexonic elements. Genetics 170 (2), 661–674. 10.1534/genetics.104.039701 15802507 PMC1450422

[B24] Bushby K. Finkel R. Wong B. Barohn R. Campbell C. Comi G. P. (2014). Ataluren treatment of patients with nonsense mutation dystrophinopathy. Muscle Nerve 50 (4), 477–487. 10.1002/mus.24332 25042182 PMC4241581

[B25] Ceco E. Bogdanovich S. Gardner B. Miller T. DeJesus A. Ju E. (2014). Targeting latent TGFβ release in muscular dystrophy. Sci. Transl. Med. 6 (259), 259ra144. 10.1126/scitranslmed.3010018 PMC4337885 25338755

[B26] Charleston J. S. Schnell F. J. Dworzak J. Donoghue C. Lewis S. Chen L. (2018). Eteplirsen treatment for Duchenne muscular dystrophy: exon skipping and dystrophin production. Neurology 90 (24), e2146–e2154. 10.1212/WNL.0000000000005680 29752304

[B27] Chen M. Wang L. Li Y. Chen Y. Zhang H. Zhu Y. (2020). Genetic modifiers of Duchenne muscular dystrophy in Chinese patients. Front. Neurol. 11, 721. 10.3389/fneur.2020.00721 32849198 PMC7403400

[B28] Choi E. Koo T. (2021). CRISPR technologies for the treatment of Duchenne muscular dystrophy. Mol. Ther. 29 (11), 3179–3191. 10.1016/j.ymthe.2021.04.002 33823301 PMC8571109

[B29] Ciafaloni E. Fox D. J. Pandya S. Westfield C. P. Puzhankara S. Romitti P. A. (2009). Delayed diagnosis in duchenne muscular dystrophy: data from the muscular dystrophy surveillance, tracking, and research network (MD STARnet). J. Pediatr. 155 (3), 380–385. 10.1016/j.jpeds.2009.02.007 19394035 PMC5884059

[B30] Clemens P. R. Rao V. K. Connolly A. M. Harper A. D. Mah J. K. McDonald C. M. (2023). Efficacy and safety of viltolarsen in boys with Duchenne muscular dystrophy: results from the phase 2, open-label, 4-year extension study. J. Neuromuscul. Dis. 10 (3), 439–447. 10.3233/JND-221656 37005891 PMC10200237

[B31] Coratti G. Pane M. Brogna C. Ricotti V. Messina S. D'Amico A. (2021). North Star Ambulatory Assessment changes in ambulant Duchenne boys amenable to skip exons 44, 45, 51, and 53: a 3 year follow up. PLoS One 16 (6), e0253882. 10.1371/journal.pone.0253882 34170974 PMC8232423

[B32] Crisafulli S. Sultana J. Fontana A. Salvo F. Messina S. Trifiro G. (2020). Global epidemiology of Duchenne muscular dystrophy: an updated systematic review and meta-analysis. Orphanet J. Rare Dis. 15 (1), 141. 10.1186/s13023-020-01430-8 32503598 PMC7275323

[B33] Crudele J. M. Chamberlain J. S. (2018). Cas9 immunity creates challenges for CRISPR gene editing therapies. Nat. Commun. 9 (1), 3497. 10.1038/s41467-018-05843-9 30158648 PMC6115392

[B34] D'Amico A. Catteruccia M. Baranello G. Politano L. Govoni A. Previtali S. C. (2017). Diagnosis of Duchenne muscular dystrophy in Italy in the last decade: critical issues and areas for improvements. Neuromuscul. Disord. 27 (5), 447–451. 10.1016/j.nmd.2017.02.006 28262469

[B35] de Couto G. Liu W. Tseliou E. Sun B. Makkar N. Kanazawa H. (2015). Macrophages mediate cardioprotective cellular postconditioning in acute myocardial infarction. J. Clin. Invest. 125 (8), 3147–3162. 10.1172/JCI81321 26214527 PMC4563759

[B36] Desjardins C. A. Yao M. Hall J. O'Donnell E. Venkatesan R. Spring S. (2022). Enhanced exon skipping and prolonged dystrophin restoration achieved by TfR1-targeted delivery of antisense oligonucleotide using FORCE conjugation in mdx mice. Nucleic Acids Res. 50 (20), 11401–11414. 10.1093/nar/gkac641 35944903 PMC9723632

[B37] Doorenweerd N. Mahfouz A. van Putten M. Kaliyaperumal R. Pac T. H. Hendriksen J. G. M. (2017). Timing and localization of human dystrophin isoform expression provide insights into the cognitive phenotype of Duchenne muscular dystrophy. Sci. Rep. 7 (1), 12575. 10.1038/s41598-017-12981-5 28974727 PMC5626779

[B38] Duan D. Goemans N. Takeda S. Mercuri E. Aartsma-Rus A. (2021). Duchenne muscular dystrophy. Nat. Rev. Dis. Prim. 7 (1), 13. 10.1038/s41572-021-00248-3 33602943 PMC10557455

[B39] Dwianingsih E. K. Malueka R. G. Nishida A. Itoh K. Lee T. Yagi M. (2014). A novel splicing silencer generated by DMD exon 45 deletion junction could explain upstream exon 44 skipping that modifies dystrophinopathy. J. Hum. Genet. 59 (8), 423–429. 10.1038/jhg.2014.36 24871807

[B40] Ebrahimzadeh-Vesal R. Teymoori A. Azimi-Nezhad M. Hosseini F. S. (2018). Next generation sequencing approach to molecular diagnosis of Duchenne muscular dystrophy; identification of a novel mutation. Gene 644, 1–3. 10.1016/j.gene.2017.12.009 29246534

[B41] Elangkovan N. Dickson G. (2021). Gene therapy for Duchenne muscular dystrophy. J. Neuromuscul. Dis. 8 (s2), S303–S316. 10.3233/JND-210678 34511510 PMC8673537

[B42] European Medicines Agency (EMA) (2024). EMA confirms recommendation for non-renewal of authorisation of Duchenne muscular dystrophy medicine Translarna. Available at: https://www.ema.europa.eu/en/news/ema-confirms-recommendation-non-renewal-authorisation-duchenne-muscular-dystrophy-medicine-translarna.

[B43] Enkhjargal S. Sugahara K. Khaledian B. Nagasaka M. Inagaki H. Kurahashi H. (2023). Antisense oligonucleotide induced pseudoexon skipping and restoration of functional protein for Fukuyama muscular dystrophy caused by a deep-intronic variant. Hum. Mol. Genet. 32 (8), 1301–1312. 10.1093/hmg/ddac286 36426838

[B44] European Medicines Agency (EMA) (2023). EMA recommends non-renewal of authorisation of Duchenne muscular dystrophy medicine Translarna. Available at: https://bit.ly/45AaJpy.

[B45] Ewaisha R. Anderson K. S. (2023). Immunogenicity of CRISPR therapeutics-Critical considerations for clinical translation. Front. Bioeng. Biotechnol. 11, 1138596. 10.3389/fbioe.2023.1138596 36873375 PMC9978118

[B46] Ferreiro V. Giliberto F. Muniz G. M. Francipane L. Marzese D. M. Mampel A. (2009). Asymptomatic Becker muscular dystrophy in a family with a multiexon deletion. Muscle Nerve 39 (2), 239–243. 10.1002/mus.21193 19012301

[B47] Finkel R. S. Flanigan K. M. Wong B. Bonnemann C. Sampson J. Sweeney H. L. (2013). Phase 2a study of ataluren-mediated dystrophin production in patients with nonsense mutation Duchenne muscular dystrophy. PLoS One 8 (12), e81302. 10.1371/journal.pone.0081302 24349052 PMC3859499

[B48] Flanigan K. M. Ceco E. Lamar K. M. Kaminoh Y. Dunn D. M. Mendell J. R. (2013). LTBP4 genotype predicts age of ambulatory loss in Duchenne muscular dystrophy. Ann. Neurology 73 (4), 481–488. 10.1002/ana.23819 PMC4106425 23440719

[B49] Flanigan K. M. Dunn D. M. von Niederhausern A. Howard M. T. Mendell J. Connolly A. (2009). DMD Trp3X nonsense mutation associated with a founder effect in North American families with mild Becker muscular dystrophy. Neuromuscul. Disord. 19 (11), 743–748. 10.1016/j.nmd.2009.08.010 19793655 PMC3142924

[B50] Fortunato F. Farne M. Ferlini A. (2021). The DMD gene and therapeutic approaches to restore dystrophin. Neuromuscul. Disord. 31 (10), 1013–1020. 10.1016/j.nmd.2021.08.004 34736624

[B51] Frank D. E. Schnell F. J. Akana C. El-Husayni S. H. Desjardins C. A. Morgan J. (2020). Increased dystrophin production with golodirsen in patients with Duchenne muscular dystrophy. Neurology 94 (21), e2270–e2282. 10.1212/WNL.0000000000009233 32139505 PMC7357297

[B52] Gazzoli I. Pulyakhina I. Verwey N. E. Ariyurek Y. Laros J. F. t Hoen P. A. (2016). Non-sequential and multi-step splicing of the dystrophin transcript. RNA Biol. 13 (3), 290–305. 10.1080/15476286.2015.1125074 26670121 PMC4829307

[B53] Gibbs E. M. Barthelemy F. Douine E. D. Hardiman N. C. Shieh P. B. Khanlou N. (2019). Large in-frame 5' deletions in DMD associated with mild Duchenne muscular dystrophy: two case reports and a review of the literature. Neuromuscul. Disord. 29 (11), 863–873. 10.1016/j.nmd.2019.09.009 31672265 PMC7092699

[B54] Grages S. M. Bell M. Berlau D. J. (2020). New and emerging pharmacotherapy for duchenne muscular dystrophy: a focus on synthetic therapeutics. Expert Opin. Pharmacother. 21 (7), 841–851. 10.1080/14656566.2020.1732350 32133879

[B55] Gualandi F. Rimessi P. Trabanelli C. Spitali P. Neri M. Patarnello T. (2006). Intronic breakpoint definition and transcription analysis in DMD/BMD patients with deletion/duplication at the 5' mutation hot spot of the dystrophin gene. Gene 370, 26–33. 10.1016/j.gene.2005.11.002 16439068

[B56] Gurvich O. L. Tuohy T. M. Howard M. T. Finkel R. S. Medne L. Anderson C. B. (2008). DMD pseudoexon mutations: splicing efficiency, phenotype, and potential therapy. Ann. Neurol. 63 (1), 81–89. 10.1002/ana.21290 18059005

[B57] Happi Mbakam C. Lamothe G. Tremblay G. Tremblay J. P. (2022). CRISPR-Cas9 gene therapy for duchenne muscular dystrophy. Neurotherapeutics 19 (3), 931–941. 10.1007/s13311-022-01197-9 35165856 PMC9294086

[B58] Hogarth M. W. Houweling P. J. Thomas K. C. Gordish-Dressman H. Bello L. (2017). Evidence for ACTN3 as a genetic modifier of Duchenne muscular dystrophy. Nat. Commun. 8, 14143. 10.1038/ncomms14143 28139640 PMC5290331

[B59] Hoy S. M. Scott L. J. Plosker G. L. (2023). Tinzaparin sodium: a review of its use in the prevention and treatment of deep vein thrombosis and pulmonary embolism, and in the prevention of clotting in the extracorporeal circuit during haemodialysis. Drugs 70, 1319–1347. 10.2165/11203710-000000000-00000 20568836

[B60] Humbertclaude V. Hamroun D. Bezzou K. Berard C. Boespflug-Tanguy O. Bommelaer C. (2012). Motor and respiratory heterogeneity in Duchenne patients: implication for clinical trials. Eur. J. Paediatr. Neurol. 16 (2), 149–160. 10.1016/j.ejpn.2011.07.001 21920787

[B61] Keegan N. P. (2020). Pseudoexons of the DMD gene. J. Neuromuscul. Dis. 7 (2), 77–95. 10.3233/JND-190431 32176650 PMC7175933

[B62] Kesari A. Pirra L. N. Bremadesam L. McIntyre O. Gordon E. Dubrovsky A. L. (2008). Integrated DNA, cDNA, and protein studies in Becker muscular dystrophy show high exception to the reading frame rule. Hum. Mutat. 29 (5), 728–737. 10.1002/humu.20722 18348289

[B63] Koeks Z. Bladen C. L. Salgado D. van Zwet E. Pogoryelova O. McMacken G. (2017). Clinical outcomes in duchenne muscular dystrophy: a study of 5345 patients from the TREAT-NMD DMD global database. J. Neuromuscul. Dis. 4 (4), 293–306. 10.3233/JND-170280 29125504 PMC5701764

[B64] Kosac A. Pesovic J. Radenkovic L. Brkusanin M. Radovanovic N. Djurisic M. (2022). LTBP4, SPP1, and CD40 variants: genetic modifiers of Duchenne muscular dystrophy analyzed in Serbian patients. Genes (Basel). 13 (8), 1385. 10.3390/genes13081385 36011296 PMC9407083

[B65] Lek A. Wong B. Keeler A. Blackwood M. Ma K. Huang S. Unexpected death of a Duchenne muscular dystrophy patient in an N-of-1 Trial of rAAV9-delivered CRISPR-transactivator. medRxiv. 2023.

[B66] Lim K. R. Q. Nguyen Q. Yokota T. (2020). Genotype-phenotype correlations in duchenne and becker muscular dystrophy patients from the Canadian neuromuscular disease registry. J. Pers. Med. 10 (4), 241. 10.3390/jpm10040241 33238405 PMC7712074

[B67] Lim K. R. Q. Woo S. Melo D. Huang Y. Dzierlega K. Shah M. N. A. (2022). Development of DG9 peptide-conjugated single- and multi-exon skipping therapies for the treatment of Duchenne muscular dystrophy. Proc. Natl. Acad. Sci. U. S. A. 119 (9), e2112546119. 10.1073/pnas.2112546119 35193974 PMC8892351

[B68] Mackenzie S. J. Nicolau S. Connolly A. M. Mendell J. R. (2021). Therapeutic approaches for Duchenne muscular dystrophy: old and new. Semin. Pediatr. Neurol. 37, 100877. 10.1016/j.spen.2021.100877 33892842

[B69] Magri F. Govoni A. D'Angelo M. G. Del Bo R. Ghezzi S. Sandra G. (2011). Genotype and phenotype characterization in a large dystrophinopathic cohort with extended follow-up. J. Neurol. 258 (9), 1610–1623. 10.1007/s00415-011-5979-z 21399986

[B70] Markati T. Oskoui M. Farrar M. A. Duong T. Goemans N. Servais L. (2022). Emerging therapies for Duchenne muscular dystrophy. Lancet Neurol. 21, 814–829. 10.1016/S1474-4422(22)00125-9 35850122

[B71] McDonald C. M. Campbell C. Torricelli R. E. Finkel R. S. Flanigan K. M. Goemans N. (2017). Ataluren in patients with nonsense mutation Duchenne muscular dystrophy (ACT DMD): a multicentre, randomised, double-blind, placebo-controlled, phase 3 trial. Lancet 390 (10101), 1489–1498. 10.1016/S0140-6736(17)31611-2 28728956

[B72] McDonald C. M. Marban E. Hendrix S. Hogan N. Ruckdeschel Smith R. Eagle M. (2022). Repeated intravenous cardiosphere-derived cell therapy in late-stage Duchenne muscular dystrophy (HOPE-2): a multicentre, randomised, double-blind, placebo-controlled, phase 2 trial. Lancet 399 (10329), 1049–1058. 10.1016/S0140-6736(22)00012-5 35279258

[B73] McDonald C. M. Shieh P. B. Abdel-Hamid H. Z. Connolly A. M. Ciafaloni E. Wagner K. R. (2021). Open-label evaluation of eteplirsen in patients with Duchenne muscular dystrophy amenable to exon 51 skipping: PROMOVI Trial. J. Neuromuscul. Dis. 8 (6), 989–1001. 10.3233/JND-210643 34120909 PMC8673535

[B74] McDonald C. M. Wu S. Gulati S. Komaki H. Escobar R. E. Kostera-Pruszczyk A. (2023). Safety and efficacy of ataluren in nmDMD patients from study 041, a phase 3, randomized, double-blind, placebo-controlled trial (PL5.001). Neurology 100 (17), 2374. 10.1212/wnl.0000000000202505

[B75] McGreevy J. W. Hakim C. H. McIntosh M. A. Duan D. (2015). Animal models of Duchenne muscular dystrophy: from basic mechanisms to gene therapy. Dis. Model Mech. 8 (3), 195–213. 10.1242/dmm.018424 25740330 PMC4348559

[B76] Mendell J. R. Sahenk Z. Lehman K. J. Lowes L. P. Reash N. F. Iammarino M. A. (2023). Long-term safety and functional outcomes of delandistrogene moxeparvovec gene therapy in patients with Duchenne muscular dystrophy: a phase 1/2a nonrandomized trial. Muscle Nerve. 10.1002/mus.27955 37577753

[B77] Mercuri E. Muntoni F. Osorio A. N. Tulinius M. Buccella F. Morgenroth L. P. (2020). Safety and effectiveness of ataluren: comparison of results from the STRIDE registry and CINRG DMD natural history study. J. Comp. Eff. Res. 9 (5), 341–360. 10.2217/cer-2019-0171 31997646 PMC7610147

[B78] Monaco A. P. Bertelson C. J. Liechti-Gallati S. Moser H. Kunkel L. M. (1988). An explanation for the phenotypic differences between patients bearing partial deletions of the DMD locus. Genomics 2 (1), 90–95. 10.1016/0888-7543(88)90113-9 3384440

[B79] Muntoni F. Gobbi P. Sewry C. Sherratt T. Taylor J. Sandhu S. K. (1994). Deletions in the 5' region of dystrophin and resulting phenotypes. J. Med. Genet. 31 (11), 843–847. 10.1136/jmg.31.11.843 7853367 PMC1016656

[B80] Muntoni F. Signorovitch J. Sajeev G. Lane H. Jenkins M. Dieye I. (2023). DMD genotypes and motor function in Duchenne muscular dystrophy: a multi-institution meta-analysis with implications for clinical trials. Neurology 100 (15), e1540–e1554. 10.1212/WNL.0000000000201626 36725339 PMC10103111

[B81] Muntoni F. Torelli S. Ferlini A. (2003). Dystrophin and mutations: one gene, several proteins, multiple phenotypes. Lancet Neurol. 2 (12), 731–740. 10.1016/s1474-4422(03)00585-4 14636778

[B82] Neri M. Rossi R. Trabanelli C. Mauro A. Selvatici R. Falzarano M. S. (2020). The genetic landscape of dystrophin mutations in Italy: a nationwide study. Front. Genet. 11, 131. 10.3389/fgene.2020.00131 32194622 PMC7063120

[B83] Norcia G. Lucibello S. Coratti G. Onesimo R. Pede E. Ferrantini G. (2021). Early gross motor milestones in Duchenne muscular dystrophy. J. Neuromuscul. Dis. 8 (4), 453–456. 10.3233/JND-210640 33935100 PMC8385509

[B84] Orso M. Migliore A. Polistena B. Russo E. Gatto F. Monterubbianesi M. (2023). Duchenne muscular dystrophy in Italy: a systematic review of epidemiology, quality of life, treatment adherence, and economic impact. PLoS One 18 (6), e0287774. 10.1371/journal.pone.0287774 37368924 PMC10298760

[B85] Pagel C. N. Wasgewatte Wijesinghe D. K. Taghavi Esfandouni N. Mackie E. J. (2014). Osteopontin, inflammation and myogenesis: influencing regeneration, fibrosis and size of skeletal muscle. J. Cell Commun. Signal. 8 (2), 95–103. 10.1007/s12079-013-0217-3 24318932 PMC4063988

[B86] Pane M. Mazzone E. S. Sormani M. P. Messina S. Vita G. L. Fanelli L. (2014). 6 Minute Walk Test in Duchenne MD patients with different mutations: 12 month changes. PLoS One 9 (1), e83400. 10.1371/journal.pone.0083400 24421885 PMC3885414

[B87] Pegoraro E. Hoffman E. P. Piva L. Gavassini B. F. Cagnin S. Ermani M. (2011). SPP1 genotype is a determinant of disease severity in Duchenne muscular dystrophy. Neurology 76 (3), 219–226. 10.1212/WNL.0b013e318207afeb 21178099 PMC3034396

[B88] Ricotti V. Mandy W. P. L. Scoto M. Pane M. Deconinck N. Messina S. (2016). Neurodevelopmental, emotional, and behavioural problems in Duchenne muscular dystrophy in relation to underlying dystrophin gene mutations. Dev. Med. Child Neurology 58 (1), 77–84. 10.1111/dmcn.12922 26365034

[B89] Rimessi P. Fabris M. Bovolenta M. Bassi E. Falzarano S. Gualandi F. (2010). Antisense modulation of both exonic and intronic splicing motifs induces skipping of a DMD pseudo-exon responsible for x-linked dilated cardiomyopathy. Hum. Gene Ther. 21 (9), 1137–1146. 10.1089/hum.2010.010 20486769

[B90] Ryder S. Leadley R. M. Armstrong N. Westwood M. de Kock S. Butt T. (2017). The burden, epidemiology, costs and treatment for Duchenne muscular dystrophy: an evidence review. Orphanet J. Rare Dis. 12 (1), 79. 10.1186/s13023-017-0631-3 28446219 PMC5405509

[B91] Salari N. Fatahi B. Valipour E. Kazeminia M. Fatahian R. Kiaei A. (2022). Global prevalence of Duchenne and Becker muscular dystrophy: a systematic review and meta-analysis. J. Orthop. Surg. Res. 17 (1), 96. 10.1186/s13018-022-02996-8 35168641 PMC8848641

[B92] Schwartz C. E. Jackson S. Valentine J. Miller N. Lowes L. Edwards D. (2023). Toward patient-centered treatment goals for duchenne muscular dystrophy: insights from the "Your Voice" study. Orphanet J. Rare Dis. 18 (1), 90. 10.1186/s13023-023-02674-w 37081508 PMC10116803

[B93] Segarra-Casas A. Dominguez-Gonzalez C. Hernandez-Lain A. Sanchez-Calvin M. T. Camacho A. Rivas E. (2023). Genetic diagnosis of Duchenne and Becker muscular dystrophy through mRNA analysis: new splicing events. J. Med. Genet. 60 (6), 615–619. 10.1136/jmg-2022-108828 36535754 PMC10313949

[B94] Sheikh O. Yokota T. (2020). Advances in genetic characterization and genotype-phenotype correlation of Duchenne and Becker muscular dystrophy in the personalized medicine era. J. Pers. Med. 10 (3), 111. 10.3390/jpm10030111 32899151 PMC7565713

[B95] Sibley C. R. Emmett W. Blazquez L. Faro A. Haberman N. Briese M. (2015). Recursive splicing in long vertebrate genes. Nature 521 (7552), 371–375. 10.1038/nature14466 25970246 PMC4471124

[B96] Spitali P. Zaharieva I. Bohringer S. Hiller M. Chaouch A. Roos A. (2020). TCTEX1D1 is a genetic modifier of disease progression in Duchenne muscular dystrophy. Eur. J. Hum. Genet. 28 (6), 815–825. 10.1038/s41431-019-0563-6 31896777 PMC7253478

[B97] Stockley T. L. Akber S. Bulgin N. Ray P. N. (2006). Strategy for comprehensive molecular testing for Duchenne and Becker muscular dystrophies. Genet. Test. 10 (4), 229–243. 10.1089/gte.2006.10.229 17253928

[B98] Taylor P. J. Maroulis S. Mullan G. L. Pedersen R. L. Baumli A. Elakis G. (2007). Measurement of the clinical utility of a combined mutation detection protocol in carriers of Duchenne and Becker muscular dystrophy. J. Med. Genet. 44 (6), 368–372. 10.1136/jmg.2006.047464 17259292 PMC2740880

[B99] Thomas S. Conway K. M. Fapo O. Street N. Mathews K. D. Mann J. R. (2022). Time to diagnosis of duchenne muscular dystrophy remains unchanged: findings from the muscular dystrophy surveillance, tracking, and research network. Muscle Nerve 66 (2), 193–197. 10.1002/mus.27532 35312090 PMC9308714

[B100] Torella A. Zanobio M. Zeuli R. Del Vecchio Blanco F. Savarese M. Giugliano T. (2020). The position of nonsense mutations can predict the phenotype severity: a survey on the DMD gene. PLoS One 15 (8), e0237803. 10.1371/journal.pone.0237803 32813700 PMC7437896

[B101] Tseliou E. Fouad J. Reich H. Slipczuk L. de Couto G. Aminzadeh M. (2015). Fibroblasts rendered antifibrotic, antiapoptotic, and angiogenic by priming with cardiosphere-derived extracellular membrane vesicles. J. Am. Coll. Cardiol. 66 (6), 599–611. 10.1016/j.jacc.2015.05.068 26248985 PMC4593504

[B102] Tuffery-Giraud S. Saquet C. Chambert S. Claustres M. (2003). Pseudoexon activation in the DMD gene as a novel mechanism for Becker muscular dystrophy. Hum. Mutat. 21 (6), 608–614. 10.1002/humu.10214 12754707

[B103] Uaesoontrachoon K. Wasgewatte Wijesinghe D. K. Mackie E. J. Pagel C. N. (2012). Osteopontin deficiency delays inflammatory infiltration and the onset of muscle regeneration in a mouse model of muscle injury. Dis. Model Mech. 6 (1), 197–205. 10.1242/dmm.009993 22917925 PMC3529351

[B104] U. S. Food and Drug Administration (FDA) 2023. ELEVIDYS (delandistrogene moxeparvovec-rokl) suspension for intravenous infusion. Highlights of prescribing information. Available at: https://www.acessdata.fda.gov, https://www.ema.europa.eu/Accessed 12 September 2023.

[B105] van den Bergen J. C. Ginjaar H. B. Niks E. H. Aartsma-Rus A. Verschuuren J. J. (2014). Prolonged ambulation in Duchenne patients with a mutation amenable to exon 44 skipping. J. Neuromuscul. Dis. 1 (1), 91–94. 10.3233/jnd-140002 27858662

[B106] Van Den Bergen J. C. Hiller M. Böhringer S. Vijfhuizen L. Ginjaar H. B. Chaouch A. (2015). Validation of genetic modifiers for Duchenne muscular dystrophy: a multicentre study assessing SPP1 and LTBP4 variants. J. Neurol. Neurosurg. Psychiatry 86 (10), 1060–1065. 10.1136/jnnp-2014-308409 25476005 PMC4602257

[B107] Vaz-Drago R. Custodio N. Carmo-Fonseca M. (2017). Deep intronic mutations and human disease. Hum. Genet. 136 (9), 1093–1111. 10.1007/s00439-017-1809-4 28497172

[B108] Vetrone S. A. Montecino-Rodriguez E. Kudryashova E. Kramerova I. Hoffman E. P. Liu S. D. (2009). Osteopontin promotes fibrosis in dystrophic mouse muscle by modulating immune cell subsets and intramuscular TGF-beta. J. Clin. Invest. 119 (6), 1583–1594. 10.1172/JCI37662 19451692 PMC2689112

[B109] Vianello S. Pantic B. Fusto A. Bello L. Galletta E. Borgia D. (2017). SPP1 genotype and glucocorticoid treatment modify osteopontin expression in Duchenne muscular dystrophy cells. Hum. Mol. Genet. 26 (17), 3342–3351. 10.1093/hmg/ddx218 28595270

[B110] Viggiano E. Picillo E. Passamano L. Onore M. E. Piluso G. Scutifero M. (2023). Spectrum of genetic variants in the dystrophin gene: a single centre retrospective analysis of 750 Duchenne and Becker patients from Southern Italy. Genes (Basel). 14 (1), 214. 10.3390/genes14010214 36672955 PMC9859256

[B111] Vo A. H. McNally E. M. (2015). Modifier genes and their effect on Duchenne muscular dystrophy. Curr. Opin. Neurol. 28 (5), 528–534. 10.1097/WCO.0000000000000240 26263473 PMC4591871

[B112] Volk A. E. Kubisch C. (2017). The rapid evolution of molecular genetic diagnostics in neuromuscular diseases. Curr. Opin. Neurol. 30 (5), 523–528. 10.1097/WCO.0000000000000478 28665809

[B113] Wagner K. R. Kuntz N. L. Koenig E. East L. Upadhyay S. Han B. (2021). Safety, tolerability, and pharmacokinetics of casimersen in patients with Duchenne muscular dystrophy amenable to exon 45 skipping: a randomized, double-blind, placebo-controlled, dose-titration trial. Muscle Nerve 64 (3), 285–292. 10.1002/mus.27347 34105177 PMC9290993

[B114] Waldrop M. A. Moore S. A. Mathews K. D. Darbro B. W. Medne L. Finkel R. (2022). Intron mutations and early transcription termination in Duchenne and Becker muscular dystrophy. Hum. Mutat. 43 (4), 511–528. 10.1002/humu.24343 35165973 PMC9901284

[B115] Wang R. T. Barthelemy F. Martin A. S. Douine E. D. Eskin A. Lucas A. (2018). DMD genotype correlations from the Duchenne Registry: endogenous exon skipping is a factor in prolonged ambulation for individuals with a defined mutation subtype. Hum. Mutat. 39 (9), 1193–1202. 10.1002/humu.23561 29907980 PMC6175390

[B116] Weiss R. B. Vieland V. J. Dunn D. M. Kaminoh Y. Flanigan K. M., (2018). Long-range genomic regulators of THBS1 and LTBP4 modify disease severity in Duchenne muscular dystrophy. Ann. Neurology 84 (2), 234–245. 10.1002/ana.25283 PMC6168392 30014611

[B117] Winnard A. V. Mendell J. R. Prior T. W. Florence J. Burghes A. H. (1995). Frameshift deletions of exons 3-7 and revertant fibers in Duchenne muscular dystrophy: mechanisms of dystrophin production. Am. J. Hum. Genet. 56 (1), 158–166. 7825572 PMC1801338

[B118] Yao S. Chen Z. Yu Y. Zhang N. Jiang H. Zhang G. (2021). Current pharmacological strategies for Duchenne muscular dystrophy. Front. Cell Dev. Biol. 9, 689533. 10.3389/fcell.2021.689533 34490244 PMC8417245

[B119] Zaidman C. M. Proud C. M. McDonald C. M. Lehman K. J. Goedeker N. L. Mason S. (2023). Delandistrogene moxeparvovec gene therapy in ambulatory patients (aged ≥4 to <8 years) with Duchenne muscular dystrophy: 1-year interim results from study srp-9001-103 (ENDEAVOR). Ann Neurol. 10.1002/ana.26755 37539981

[B120] Zambon A. A. Waldrop M. A. Alles R. Weiss R. B. Conroy S. Moore-Clingenpeel M. (2022). Phenotypic spectrum of dystrophinopathy due to Duchenne muscular dystrophy exon 2 duplications. Neurology 98 (7), e730–e738. 10.1212/WNL.0000000000013246 34937785 PMC8865888

[B121] Zaum A. K. Stuve B. Gehrig A. Kolbel H. Schara U. Kress W. (2017). Deep intronic variants introduce DMD pseudoexon in patient with muscular dystrophy. Neuromuscul. Disord. 27 (7), 631–634. 10.1016/j.nmd.2017.04.003 28495050

